# Underlying Mechanisms and Treatment of Cellular Senescence-Induced Biological Barrier Interruption and Related Diseases

**DOI:** 10.14336/AD.2023.0621

**Published:** 2024-04-01

**Authors:** Ruize Sun, Juan Feng, Jue Wang

**Affiliations:** Department of Neurology, Shengjing Hospital, Affiliated Hospital of China Medical University, Shenyang, China

**Keywords:** mechanisms, treatment, cellular senescence, biological barrier interruption, related diseases

## Abstract

Given its increasing prevalence, aging is of great concern to researchers worldwide. Cellular senescence is a physiological or pathological cellular state caused by aging and a prominent risk factor for the interruption of the integrity and functionality of human biological barriers. Health barriers play an important role in maintaining microenvironmental homeostasis within the body. The senescence of barrier cells leads to barrier dysfunction and age-related diseases. Cellular senescence has been reported to be a key target for the prevention of age-related barrier diseases, including Alzheimer's disease, Parkinson's disease, age-related macular degeneration, diabetic retinopathy, and preeclampsia. Drugs such as metformin, dasatinib, quercetin, BCL-2 inhibitors, and rapamycin have been shown to intervene in cellular senescence and age-related diseases. In this review, we conclude that cellular senescence is involved in age-related biological barrier impairment. We further outline the cellular pathways and mechanisms underlying barrier impairment caused by cellular senescence and describe age-related barrier diseases associated with senescent cells. Finally, we summarize the currently used anti-senescence pharmacological interventions and discuss their therapeutic potential for preventing age-related barrier diseases.

## 1. Introduction

Cellular senescence is a state of stable cell cycle arrest that occurs in response to various stressors. Cellular senescence refers to replicative senescence in normal human diploid fibroblasts with limited replicative capacity, which results in telomere shortening or dysfunction induced by various pathophysiological forms of stress [^1^]. Although senescent cells exhibit irreversible proliferative arrest, they remain metabolically active. Senescent cells secrete a series of pro-inflammatory and hydrolytic factors as part of the senescence-associated secretory phenotype (SASP), which inhibits the onset of apoptosis, accelerates senescence and leads to the development of age-related diseases. SASP is also one of the hallmarks of cellular senescence, in addition to extended cell cycle arrest, transcriptional changes, macromolecular damage, and metabolic dysregulation ^[^2^]^. Senescence is a highly dynamic multistep process during which senescent cells evolve and diversify. After a long period of culture, senescent cells continue to evolve to a stage known as "deep" or "late" senescence.

Many biological barriers exist in the human body and function as immune-enhanced boundaries that separate tissues and organs from the environment to ensure their sterility and stability. These physiological barriers are usually highly selective semi-permeable barriers that restrict the entry of blood cells and plasma components into the corresponding organ tissues, facilitate the influx of essential nutrients, and mediate the efflux of metabolic waste products. These processes provide an optimal environment for tissues and organs. Biological barriers are regulated and adapted to the environment depending on their structure and function. Different biological barriers such as the blood-brain barrier (BBB), blood-retinal barrier (BRB), intestinal barrier (IB), placental barrier (PB), and blood-testis barrier (BTB) have different immune strategies to maintain homeostasis in the internal environment. These strategies are called “barrier immunity” and help maintain the integrity and functionality of the biological barriers.

Biological barriers are continuously exposed to various stimuli that affect barrier formation and function. The barriers are also the first line of defense against pathogens. Disruptions in barrier integrity are closely associated with disease onset. Cellular senescence is a defense mechanism that prevents cellular damage. Since senescent cells accumulate with age, we hypothesize that cellular senescence contributes to age-related barrier dysfunction and diseases. Researchers have found that senescent cells may drive age-related biological barrier dysfunction through two mechanisms: loss of function (within the barrier region where T cells are expressed; senescent cells reduce the ability of T cells to perform immune regulatory activity) and the effects induced by SASP factors (senescent cells secrete inflammatory cytokines, chemokines, growth factors, and proteases that cause cellular damage to surrounding cells, thereby accelerating senescent damage to the barrier). Low levels of SASP are considered beneficial since they stimulate barrier repair and enhance immune surveillance; however, the presence of large amounts of SASP factors in age-related biological barriers may disrupt barrier structure and function by interrupting intercellular signal transduction and maintaining a pro-inflammatory environment [3^]^. Thus, the removal of senescent cells may reduce the onset of aged-related diseases [4], such as Alzheimer's disease, Parkinson's disease, age-related macular degeneration, diabetic retinopathy, and preeclampsia. Currently, there are no effective treatments for these diseases. The available pharmacological interventions are aimed at reducing oxidative stress, DNA methylation, and the inflammatory response.

This review presents the results of recent studies on pathological changes, underlying mechanisms, and related predisposing diseases and their interventions in barrier damage caused by cellular senescence. Several drugs that are currently used to intervene in the cellular senescence process are also discussed. This review aims to lay a theoretical foundation for reversing cellular senescence, cellular senescence-induced biological barrier disruption, and related diseases.

## 2. Aging and Cellular Senescence

With the increases in global aging trends, aging and age-related diseases are becoming a focal point in research. Senescence is a part of the cell cycle and not only participates in physiological and pathological processes but also plays an important role in aging and age-related diseases. Although aging and senescence are associated with each other, they involve different physiological processes.

### 2.1 Aging

Aging is a natural physiological process characterized by functional decline. It occurs in multiple organ systems in mammals, leading to progressive deterioration and tissue dysfunction. Aging is a risk factor for many diseases, such as cardiovascular diseases [5], dementia [6], osteoporosis, osteoarthritis [7], cancer [^8^], type 2 diabetes [9], idiopathic pulmonary fibrosis [10], and glaucoma [11]. There are three main types of physiological and pathological changes that occur during aging: primary or age-associated damage, antagonistic damage, and integrative or consequent responses to the aging phenotype [12].

### 2.2 Senescence

Senescence is a physiological process that differs from aging. Senescence is a state of stable cell cycle arrest and antagonizes cell proliferation [13]. There are two main types of senescence: replicative and premature. Replicative senescence is a stress response triggered by age-related damage, such as genomic instability, which is the main hallmark of aging. Replicative senescence prevents the physiological response of genomic instability and accumulation of DNA damage [14] and plays a physiological role in the maintenance of tissue stability during normal development. Replicative senescence is closely associated with the antagonistic characteristics of aging. For example, replicative senescence induces a decline in mitochondrial phagocytosis, which causes defects in the mitochondrial network and further limits cell proliferation and metabolic function [15]. Senescent cells further accumulate in age-related pathological sites over time, thus affecting the progressive dysfunction of the physiological tissue system.

The other type of cellular senescence is premature senescence, which is an accelerated cellular senescent cycle independent of telomere shortening [11][16]. Such senescence occurs immediately after damage, such as genotoxic stress or metabolic shock, which can be triggered by integrative conditions. The overexpression of a proto-oncogene or loss of a tumor suppressor gene in oncogenic cells can also cause premature senescence [17].

Senescent cells are usually characterized by a change in size, shape, age-related heterochromatin lesion formation [18], lipofuscin accumulation [19], DNA damage lesions [20], loss of laminin B1 [21], cellular senescent satellite-related expansion [22], differentiated embryo-chondrocyte expressed gene 1 and decoy receptor 2 expression [23], and the secretion of microRNAs, SASPs, or senescence information secretory group factors, such as cytokines, chemokines, and proteases [24][25]. These factors affect the biological microenvironment in different ways and can cause serious changes in cell metabolism to complete senescence [26]. The consumption of glycogen phosphorylase leads to glycogen accumulation, which is related to reinduced proliferation and cellular senescence [27]. In addition, telomere erosion, oncogene overexpression, reactive oxygen species (ROS)-mediated DNA dysfunction, mitochondrial dysfunction, and inflammation can drive cellular senescence [9]. These characteristics define the gold standard for cellular senescence and represent its biological characteristics. In mitogenic environments, the genes involved in cell proliferation are underexpressed [28]. Thus, cell cycle arrest is a critical characteristic for identifying all types of cellular senescence. However, several cellular mechanisms drive stable replication arrest. Cell cycle arrest in senescent cells is associated with increased levels of cell cycle inhibitors, including p16INK4a, p21CIP1, and p27. The expression levels of p19ARF, p53, and PAI-1 are increased in senescent cells and may be used as senescence markers [29].

### 2.3 Interaction Between Aging and Cellular Senescence

Although aging and senescence are independent physiological processes, interactions exist between them. Aging and age-related diseases play a crucial role in senescence. Senescence can, in turn, cause age-related complications, such as stem cell exhaustion, chronic inflammation, proteostatic dysfunction, and nutrient signaling disruption [30].

Stem cell exhaustion occurs with age, and a decline in the functional and renewable abilities of senescent stem cells can induce tissue degradation [31]. Hematopoietic stem cells (HSCs) play an important role in the development of various blood diseases. An increase in the number of senescent HSCs [32] is associated with lower immunity [33], fewer naive T and B cells [34], and lower natural killer (NK) cell activity [35]. The senescence of bone marrow mesenchymal cells (BMSCs) impairs stemness and osteogenic differentiation. The nucleosome assembly protein 1-like 2 (NAP1L2) is associated with the senescence of BMSCs and osteogenic damage. In a previous study, the screening of age-related gene expression profiles in human BMSCs showed that higher NAP1L2 expression led to restrained osteogenic differentiation and induced senescence in BMSCs [36]. With increasing age, neural stem cells (NSCs) show limited neurogenic capacity, which causes a 2-fold decrease in the number and proliferation of NSCs. Aging is also associated with increased β-galactosidase expression in NSCs [37]. Decreased renewability with age occurs in mesenchymal stem cells (MSCs) and MSC descendants [38], satellite cells [39], chondrocytes [40], and adipocytes [41], which are also associated with increased levels of senescent biomarkers [42].

Low-level inflammation is another risk factor for age-related diseases. The inflammatory response is the main extrinsic reaction in senescent cells, demonstrating a link between aging and cellular senescence [43].

Protein homeostasis or stability ensures precise control over protein synthesis, folding, conformational maintenance, and degradation [44]. Age-related protein diseases arise from a decreased ability to maintain protein stability during senescence [45]. An increase in the aggregation of misfolded proteins can lead to cardiovascular diseases [46], in which there is an abnormal deposition of multiple components of proteostasis systems in the heart and vasculature. The components of proteostatic systems include chaperones, the ubiquitin-proteasome system, and the lysosome-autophagy system [47]. Changes in age-related factors affect chaperone activity. For example, aging results in the downregulation of heat shock protein 70 (HSP70) in vascular tissues [48]. Cellular senescence results in mitochondrial dysfunction, downregulated cellular adenosine triphosphate (ATP), and the aggregation of misfolded proteins [48]. Dysfunctional autophagy is a common pathway that accelerates vascular aging and the development of age-related vascular diseases. The stimulation of autophagy (e.g., by trehalose or spermidine treatment) has been reported to reverse arterial aging [49]. Proteasome activity is further decreased in atherosclerotic plaques in elderly patients, and the ubiquitin-proteasome system plays an important role in activating the key regulators of atherogenesis and vascular inflammation [50].

Nutrient signaling is disrupted during cellular senescence and aging. mTORC1-autophagy impacts the human nutrient-sensing pathway. The age-related accumulation of pro-inflammatory senescent cells contributes to a decline in organismal fitness. Disruptions in nutrient signaling can further promote senescent cell death, which has important implications for healthy aging [51].

Although aging and senescence are two different pathophysiological processes, they can interact with each other. Aging and age-related diseases are affected by several factors that induce senescence. In addition to aging and age-related diseases, various cytokines and pathways can induce senescence, thereby aggravating the aging process. Future studies should not only distinguish senescence from aging but also explore the interactions between them and the underlying molecular mechanisms. Such developments would contribute to combating the global aging trend.

## 3. Role of Cellular Senescence in Age-Related Biological Barriers

As previously mentioned, cellular senescence plays a significant role in the development of age-related biological barriers. Various biological barriers (e.g., the BBB, BRB, BAB, PB, and BTB) in the human body have functions such as preventing foreign object invasion, secreting bactericidal substances, and antagonizing normal flora. With aging, the number and proliferation of senescent cells also increase. Because biological barriers are composed of various cells, the development and proliferation of senescent cells could bring about age-related changes in the barriers, thus affecting their integrity and function. In this section, we discuss the cellular differences caused by senescence at different age-related barriers. The function of cellular senescence in impaired barriers is further discussed to highlight the link between cellular senescence and age-related biological barriers.

### 3.1 Cellular Senescence in Age-Related Blood-Brain Barrier Disruption

The BBB is a key biological barrier in the human body. It is involved in the exchange of substances between the peripheral blood and brain parenchyma, prevents the entry of toxins and pathogens into the brain, and plays a key role in the influx and efflux of biological materials. The functional and structural integrity of the BBB is critical for protecting brain health and maintaining homeostasis in the brain microenvironment. Age-related BBB disruption has emerged as a potential risk factor for various neurological diseases. The accumulation of senescent cells in the BBB has become recognized as a critical step in age-related BBB destruction.

Recent studies have shown that the physiological effects of the BBB diminish with aging and are undermined by many age-related diseases, including Alzheimer’s disease, Parkinson’s disease, Age-related Macular Degeneration and Diabetic Retinopathy and so on [52]. Cerebrovascular endothelial cells (CECs) constitute a central part of the BBB [53]. Under biological conditions, CECs are responsible for molecular transport between the brain parenchyma and periphery. There are numerous BBB-specific transporters and receptor proteins present in the cell membranes of CECs to control the entry and exit of metabolites between cells: transcellular transport, for example. The high resistance of tight junctions (TJs) restricts material transduction between adjacent cells, such as that in paracellular transport, for instance [54]. Histopathological studies conducted on normal aging populations and populations with cerebrovascular disease have found that the processes brought about by aging, such as IgG extravasation, TJ protein changes, cerebral microhemorrhages, and microvascular endothelial dysfunction, may induce CEC dysfunction and senescence. This may lead to increased BBB permeability and BBB disruption [55]. Peripheral lymphocytes that infiltrate the cerebral vasculature can further induce inflammation and a progressive age-dependent increase in BBB permeability [56].

Pericytes are also involved in the TJs of the BBB, and their properties change with aging. The progressive loss of pericytes in aged mice has been found to aggravate BBB destruction, thus allowing intracranial microvascular degeneration, inadequate nutrient supply, neuronal dysfunction, and cognitive impairment, eventually leading to neurodegeneration [57]. It has also been found that as the number of pericytes with a senescent phenotype increases, the concentration and degradation of exogenously circulating lysosomes also increase, leading to pericyte deficiency and accelerating BBB breakdown [58].

Neurons in the brain are non-renewable, making them the most vulnerable to age-related damage [59]. Some studies have found that neurons exhibit a senescent phenotype, but it is unclear if neurons are senescent cells. Under normal conditions, neurons produce long axonal projections through neurofilaments. In contrast, neurons with a senescent phenotype have axons with wider plate sites and increased distances from the soma and exhibit a reduced intra-axonal elimination of toxins, which increases the exposure of neurons to toxins [60]. Calcium dysregulation and reduced levels of protective calcium-binding proteins occur in senescent neurons, making the neurons vulnerable to excitotoxicity and oxidative stress. These factors increase the probability that normal neuronal cells will undergo senescence [61]. Neurons in the subventricular zone (SVZ) and subgranular zone (SGZ) have regenerative capacities. Insulin-like growth factor-1 (IGF-1) and beta 2 microglobulin are negative regulators of neurogenesis, and their expression can be significantly upregulated in an aging SVZ and SGZ [62], which can cause an age-dependent neural decline at these two sites [63]. In a neuronal cell model of the senescent phenotype, it was found that increased Ca^2+^ transfer from the endoplasmic reticulum to the mitochondria played the following roles in senescence: (1) It allowed the downregulation of stored calcium, leading to age-related cognitive decline [64]; (2) mitochondrial calcium uniporter (MCU) was concomitantly upregulated, and endoplasmic reticulum contacts, a hotspot of Ca^2+^ signaling, was involved in this process [65][66]; and (3) it participated in the regulation and homeostasis of mitochondrial potential in cells with a senescent phenotype through the mechanisms of mitochondria-ER contacts (MERC) flow (control of calcium channel expression levels and the number and structure of MERCs) [67]. The expression of MCU and inositol 1,4,5-trisphosphate receptor type 2 (ITPR2) further decreased in aging neurons, resulting in increased mitochondrial calcium accumulation and accelerating senescence [68].

Age-related damage affects glial cells and neurons. Although the number of microglia increases in older brains, there is a significant decline in microglial function. Senescent microglia exhibit a buildup of lipofuscin granules and enlarged and stiffened cell bodies, and their immune capacity decreases with age [69]. Senescent microglia are also easily polarized to the M1 phenotype, with elevated expression levels of pro-inflammatory factors and hyper-responsiveness to noxious stimuli [70]. Senescent microglia are further poorly responsive to regulatory signals, such as tumor necrosis factor (TNF)-β and exhibit a reduced expression of the anti-inflammatory phenotype M2 [71]. A previous study involving the single cell sequencing of mouse microglia showed that adult mouse microglia subpopulations are less heterogeneous, while the heterogeneity of aged mouse microglia increases during senescence. The increased expression of CCL4 chemokines by senescent microglia helped in the processes of increased heterogeneity and promoting inflammation [72].

Oligodendrocytes are involved in the formation of the myelin sheaths of neuronal axons to ensure the high-speed transduction of nerve signals [73]. The degeneration of myelin sheaths in oligodendrocytes of age-related BBB indicates a marked reduction in their ability to repair and regenerate myelin sheaths, as well as the thinning of regenerating myelin sheaths, shortening of intersegmental lengths, marked reductions in conduction velocity, and extensive and irreversible demyelination [74].

Astrocytes are the cells in the brain that are the least affected by aging. The number of astrocytes does not decrease during aging [75]. The morphologies of astrocytes tend to flatten during aging [76]. The abundance of transporters, such as glutamate aspartate transporter (GLAST) and purinergic receptors, decreases with age, which accelerates age-related neuronal damage [77]. Age-related BBB dysfunction (BBBD) leads to the excessive activation of transforming growth factor (TGF)-β signaling in astrocytes, which triggers a pro-inflammatory and epileptogenic phenotype. Over time, BBBD-associated astrocytic dysfunction may lead to hippocampal and cortical neural hyperexcitability and cognitive impairment [78]. In the aging population, astrocytes with a senescent phenotype accumulate in the brain and exhibit decreased expression levels of transporters (e.g., GLAST) and purinergic receptors and the secretion of SASP factors, which can lead to neuroinflammation and neurotoxicity. Age-related neuronal damage can then accelerate. The extent of BBBD progressively increases with age and is associated with an increased risk of neurodegenerative diseases [79].

### 3.2 Cellular Senescence in Age-Related Blood-Retinal Barrier Disruption

The accumulation of senescent cells is associated with impaired BRB integrity, which may provide insights into the mechanisms underlying age-related BRB disruption. The BRB is formed by complex TJs of retinal capillary endothelial cells and retinal pigment epithelial cells, which control the movement of fluid and nutrient molecules between the ocular vascular bed and retinal tissue, prevent the free diffusion of substances and infiltration of macromolecules and potentially harmful substances into the retina, and maintain normal retinal microvascular homeostasis. The integrity of the BRB is essential for the structural and functional homeostasis of the retina. Age-related damage caused by cellular senescence can affect the integrity and function of the BRB [80]. Retinal pigment epithelial (RPE) cell senescence, the accumulation of cellular metabolic debris and protein aggregates, thickening of Bruch's membranes, and changes in extracellular matrix composition occur during age-related BRB disruption [81].

In a previous study that analyzed normal young retinas (< 45 years old), RPE cells were mostly hexagonal and evenly distributed in a single layer of cells. By contrast, in age-related retinas, some RPE cells exhibited premature senescence. The number of cells with normal hexagonal morphologies decreased, and the cell bodies were irregularly enlarged. The total number of posterior poles of RPE cells remained unchanged [82]. Retinal thickness decreased with aging. Only two or three rows of cells remained in the inner retinal layer of the severely aged retinas, and there was a significant decrease in thickness [83]. Endothelial cell loss, neuronal cell senescence, and mild microglial activation occur in normal age-related retinas [84], whereas T- or B-cell activation is uncommon [85]. ZO-1 has been reported to be absent in senescent RPE cells, suggesting that senescent RPE cells may induce TJ disruption in the BRB [86]. In cases of low-level damage to the RPE, there is an age-dependent accumulation of subretinal phagocytes [87]. The choroid becomes thinner with aging, and the densities of choroid capillaries decrease, which leads to a deficiency in BRB blood flow and reperfusion capacity and further induces vascular inflammation in the retina and choroid [88]. Retinal microglia are phagocytes that undergo age-related changes. In young individuals, the outer layer of the retina is typically free of microglia. The number of microglia increases significantly with aging and leads to age-related manifestations. During aging, dendritic axes become smaller, and dendrites become less mobile and lack motile cellular processes; thus, the responsiveness of the eyes to injury is much slower than that of young eyes. In age-related BRB disruption, microglia contact the RPE and cause a decrease in RPE-producing melanin and an increase in lipofuscin production and accumulation, thereby promoting inflammatory responses in BRBs [89]. Senescent microglia also appear to have heightened immune vigilance and may engulf live neurons or engage in excessive synaptic pruning via complement component 1q, leading to impaired synaptic connections and dysregulated phagocytosis [90][91]. During the development of age-related neurodegenerative diseases, the phagocytic activity of the RPE against apoptotic vesicles, protein aggregates, and myelin is insufficient, which may lead to the progressive accumulation of potentially toxic compounds [92].

Healthy blood vessels and intact pericytes are essential for stem cell proliferation and differentiation. Stem cells can be generated from pluripotent progenitors and Müller glial cells [93]. In aged BRBs, the loss of endothelial cells and pericytes disrupts vascular integrity and can lead to stem cell failure [94][95][96]. During eye development, retinal neurons differentiate from retinal stem cells. The number of neurons in the BRB decreases with age, leading to age-related visual acuity loss [41]. Stem cell failure due to aging further slows neuronal cell renewal in the BRB. The exposure of neurons to increased oxidative stress damage may accelerate neuronal cell apoptosis, leading to microvascular damage and BRB breakdown [97]. Retinoblastoma protein (RB) is also overexpressed during aging and regulates the expression of osteocalcin, fibronectin, ApoJ, and SM22 [98]. Retinal stem cells also differentiate into RPE cells [99]. During aging, the RPE undergoes significant morphological and functional changes. The numbers and sizes of RPE cells increase as phagocytic and lysosomal activity decreases [100][101]. The RPE regenerates photopigment molecules, such as melanin, upon exposure to light, but this ability decreases with age [102]. The reduced circulation of photopigments results in weakened intercellular interactions between the RPE and retinal photoreceptors, leading to visual impairment [34].

### 3.3 Cellular Senescence in Age-Related Intestinal Barrier Disruption

The intestinal barrier is a key physiological barrier for nutrient absorption and the maintenance of health in humans and animals. It selectively allows substances to enter the intestines and prevents access to harmful materials, which protects the host from intestinal microbes, food antigens, and toxins in the gastrointestinal tract. The integrity of the intestinal barrier is essential to ensure the proper development of each gastrointestinal cell, maintenance of immune function, and tolerance to dietary antigens and intestinal microbiota. Researchers have found that the integrity of the intestinal barrier may be affected by cellular senescence [103].

Intestinal epithelial cells are some of the most versatile epithelial cells in humans. The intestinal epithelium is involved in many complex physiological processes, including nutrient absorption, immune regulation, and microbial symbiosis. The intestinal epithelium also acts as an important physical and chemical barrier to the internal environment of the body and as an integration site in response to various physiological and pathophysiological stimuli [104]. Aging poses a significant challenge to the renewal and protection of intestinal epithelial cells. Intestinal epithelial cells in the small intestines differentiate into ciliated cells and form crypts or villous structures. Stem cells are active in crypts and constantly produce new villous epithelial cells.

Aging leads to age-related morphological and functional changes in intestinal cells, with crypts becoming shallow or even disappearing and intestinal epithelial cells losing their viability. This may affect the adaptability of stem cell proliferation and differentiation, leading to stem cell fatigue, depletions in intestinal epithelial ciliated cells that are not regularly renewed, reduced nutrient transport and absorption capacities, increased exposure to intestinal pathogens, triggered immune and inflammatory responses, and impacted epithelial homeostasis and IB function [105][106][107]. Aging *Drosophila* gastric epithelial copper cells have been found to transform into posterior midgut intestinal epithelial cells through ectopic JAK/STAT signaling activation, a decrease in gastric secretory acidic cells [108], decrease in acidification in the gastric region, changes in symbiosis from anterior midgut colonization to posterior midgut colonization [109], chronically elevated Upd inflammatory cytokine expression, the chronic activation of p38/Duox in the intestinal epithelium, chronic accumulation of ROS, and disruptions in normal Nr2 signaling, all of which can result in the improper differentiation of intestinal stem cells and poor intestinal epithelial development [110].

### 3.4 Cellular Senescence in Age-Related Placental Barriers

Cellular senescence plays a vital role in reproductive development, and the accumulation of pathologically senescent cells in the PB due to disease or other factors can easily induce reproductive failure. Reasonable and accurate cellular senescence is an important aspect of PB and embryonic development. The placental barrier is an important channel for the transfer of nutrients between the mother and fetus, a source of nutrition for fetal growth during pregnancy, and an important physical and physiological barrier that prevents harmful substances from entering the fetus and preventing the fetus from being disturbed by other external factors. The PB plays an important role in fetal and adult health [111]. Recent studies have demonstrated that the placenta has a predetermined lifespan. As the fetus grows, placental cell function declines. Researchers have referred to this change as “placental aging”. Premature placental senescence caused by disease and cellular senescence-related factors (telomere shortening, mitochondrial dysfunction, and oxidative damage) is known as “pathological placental senescence” [112]. Placental aging and senescence can cause changes in cells in the PB and limit their survival [113]. The cytotrophoblast is composed of replicated cells that continue to reproduce, whereas the syncytiotrophoblast is a cell tissue in an aging state and cannot reproduce. During embryonic implantation and the first trimester of pregnancy, NK cells acquire senescent characteristics through the upregulation of p21Cip1/War1 and pHP1. Soluble non-classical major histocompatibility complex molecules secreted by the fetal trophectoderm activate NK cells via CD158d, inducing permanent cell cycle arrest, DNA damage accumulation, and chromatin clearance. Senescent NK cells then begin to produce specific SASPs that cause neovascularization during embryonic transplantation [114]. In pregnant women with placental aging or senescence, the overcrowding of the terminal villi of the placental cell trophectoderm and narrowing of intervillous pore spaces cause intervillous hypoxia and promote mesenchymal transition [115]. Precocious stress in the syncytial trophectoderm accelerates placental senescence. When its growth has reached its limit, respiratory failure can occur in the PB [116]. Trophoblast damage interacts with endometrial cell damage to accelerate endometrial cell senescence and increase the potential for uterine metaplasia and implantation [117].

Aging placentas are prone to ROS accumulation, which may accelerate the loss of heterochromatin in the mesenchymal progenitor cell (MPC) inhibitory zone, weaken euchromatin in the active zone, switch the interface topological zone, and increase epigenetic entropy, thereby causing the nuclear deformation of MPCs, which may intensify the invasion of fetal genetic chromatin [118]. HSCs appear in the aortogonadal-midrenal region of the embryo and placenta. Aging placentas reduce cell division and the production of fetal hematopoietic stem cells, which cannot supply the fetus with sufficient blood volume [119]. Aging oocytes produced by aging ovaries in older women are usually accompanied by functional defects, such as chromosomal abnormalities [120], a diminished response to endocrine signals [121], the degeneration of red blood cell capacities [122], and interference with the molting of the uterine stroma [123]. This results in severe defects in placental formation and endothelial cell damage, making it difficult for the placenta to provide adequate nutrition for fetal growth [124] and increasing the risk of maternal pregnancy complications, such as preeclampsia [125], and fetal defects, such as congenital heart disease [126], miscarriage, and stillbirth [127]. HMGB2 levels, which are involved in maintaining 3D chromatin structures, decrease prior to the appearance of established senescence markers in umbilical vein endothelial cells (HUVECs), fetal lung fibroblasts (IMR90s), and mesenchymal stromal cells (MSCs), thereby inducing senescence [128]. In a previous study involving the single-cell sequencing of the blood-fetal barrier in elderly individuals, CDKN1A, MTOR, MYOT, and UBE2E1 were found to play key roles in the inhibition of PB senescence [129].

### 3.5 Cellular Senescence in Age-Related Blood-Testis Barriers

Senescent cells gradually accumulate in the BTB with increasing age. The BTB is one of the tightest physiological barriers in the body. Not only does the BTB consist of TJs that act as biological barriers to restrict the transport of biomolecules and ions from the basal zone to the luminal zone, but TJs also coexist and function together with ectoplasmic specificity, bridging granules, and gap junctions. BTBs further isolate luminal-dwelling germ cells from the circulatory and lymphatic systems, and together with local immunosuppression, create a unique microenvironment for the completion of meiosis and development of sperm cells into spermatozoa [130]. The growing number of senescent cells affects the integrity and function of the BTB and may lead to reproductive disorders. As individuals age, germ cells are lost in the testes, the TJs between the germinal epithelium and germ cells are weakened, expression of proteins involved in the junctions is reduced, the BTB ages and becomes thinner, and the ability to protect germ cells is weakened. This provides an opportunity for harmful substances and immune cells to cross the BTB, where germ cells are attacked, and spermatogenesis is interrupted [131].

In age-related BTBs, stem cell renewal is restricted, and normal varicose seminiferous tubules, composed of Leydig and peritubular cells, are replaced by completely degenerated germ cell-free tubules [132]. The enlarged intercellular spaces between Leydig cells are occupied by Sertoli cells [133], steroid function is reduced, hedgehog signaling, and testosterone production are reduced, peritubular cell death is increased, expression of Sertoli cells and myoblast parental imprinting genes are dysregulated [134], TJs (Sertoli-Sertoli junctions) are reduced and converted to dot junctions, and the DNA damage response of peritubular myoblasts is increased. There are also varying degrees of epithelial cell nuclei deformation and reduced expression of the endothelial gap junction protein Gja1 but unchanged endothelial cell numbers and a weak BTB [135].

**Figure 1.**
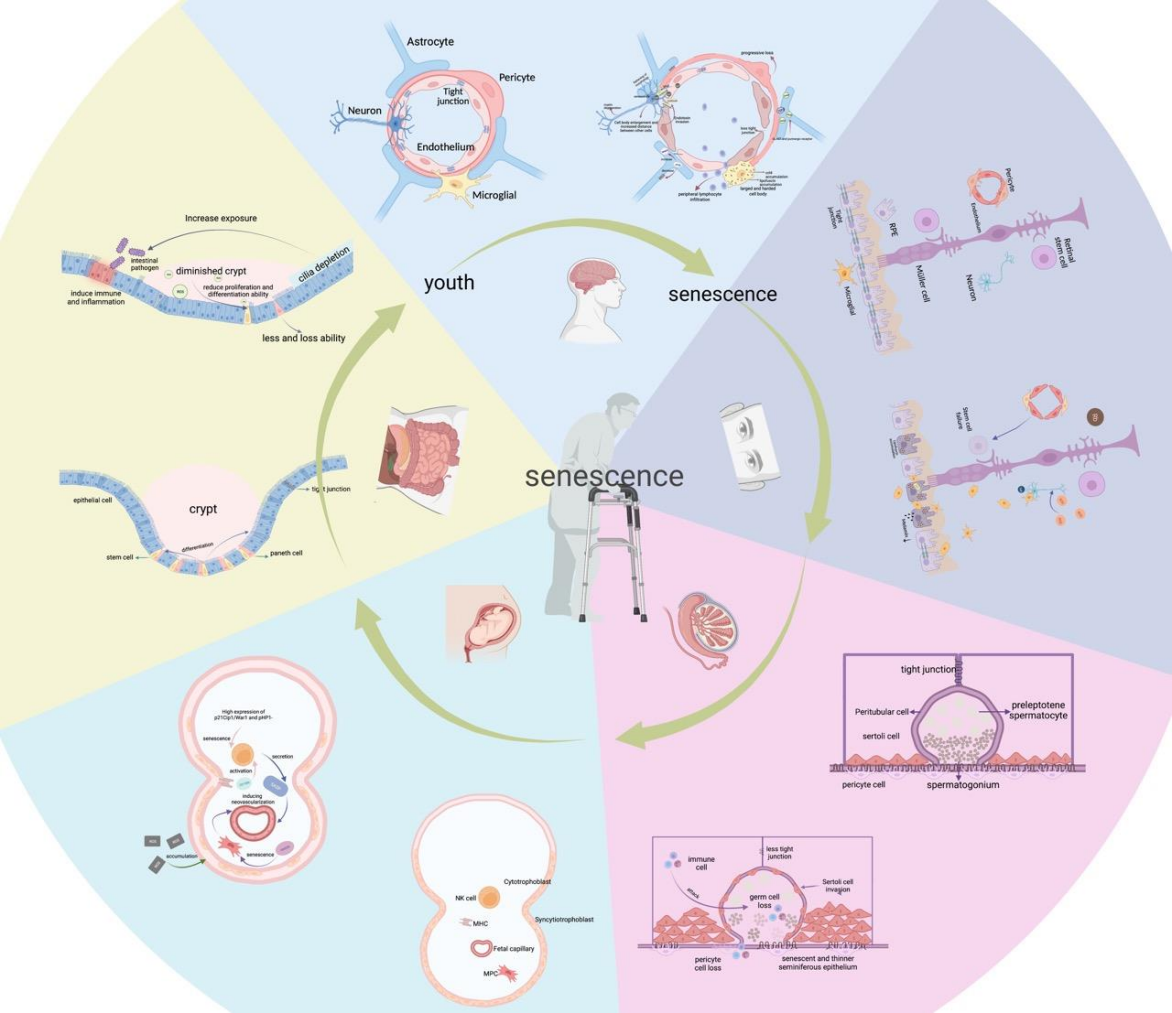
**Cellular changes and regulation of the biological barrier from youth to senescence**. **(A)** Changes in astrocytes, pericytes, endothelial cells, microglia, neurons, and secretory factors from young BBB to senescent BBB. **(B)** Changes in retinal stem cells, Müller cell, RPE, pericyte, endothelium, neuron and microglial, etc., and secretory agents from young BRB to senescent BRB. **(C)** Changes in the peritubular cell, Sertoli cell, preleptotene spermatocyte, spermatogonium, pericyte cell, etc., and secretory elements from young BTB to senescent BTB. **(D)** Changes in cytotrophoblast, syncytiotrophoblast, MPC, etc., and secretory factors from young PB to senescent PB. **(E)** Changes in the epithelial cell, stem cell, paneth cell, and some other structure or secretory components from young IB to senescent IB.

Each biological barrier plays an important role in the body. As the body ages physiologically and pathologically, the cells in each barrier change accordingly (Fig. 1). By understanding these changes, we can better understand the effects of aging on physiological barriers and be better equipped to manage these effects.

### 3.6 Mechanisms Underlying Cellular Senescence-Induced Barrier Impairment

Senescent cells play an important role in age-related barrier disruption. As senescent cells continue to experience and produce various stresses with age, they accumulate and spread through age-related biological barriers, thereby inducing barrier disruption [136]. In this section, we describe the mechanisms underlying cellular senescence-induced barrier impairment.

#### 3.6.1 Cell Structure and Cycle

Cellular senescence is closely related to biological barrier integrity and functionality, and the disruption of cellular structural integrity and cell cycle arrest are key features of age-related biological barriers [137]. Senescent cells undergo significant morphological changes. The cells and their nuclei are significantly enlarged, Golgi apparatus is prominent, cytoplasm shows vacuolization, and cytosol loses support, collapses, and tends to flatten [138][139], eventually forming senescence-associated heterochromatin foci (SAHF) on the age-related biological barrier [140]. SAHF are DNA-concentrated regions in the nuclei of senescent cells associated with transcriptional inactivation sites; the organization of their heterochromatin undergoes senescence-associated changes that inhibit the chromatin environment and prevent the transcription of growth-promoting genes, thereby stabilizing the cellular senescence state [141]. The progressive accumulation of senescent cells with cell cycle arrest in age-related barriers reduces intracellular RNA turnover and protein degradation through proteasome-mediated pathways [142], separated cell growth and cell division, and the presumed blockage of senescent cells in the G1 block, resulting in increased RNA and protein content in the senescent cells and triggering the generation and accumulation of different heterochromatic structures and SAHF formation. As biological barrier aging progresses, SAHF levels increase, which causes the acceleration of cellular senescence and affects the normal function of the barrier.

Mitochondria-associated membranes (MAMs) are platforms that enable communication between the endoplasmic reticulum and mitochondria. In senescent cells, MAM thickness is significantly increased, and cellular mitochondrial calcium uptake is impaired, making Ca^2+^ transport less efficient. Ca^2+^ inward flow is necessary to stimulate ERK1/2 activation and angiogenesis, and the Ca^2+^ concentrations inside and outside the cell are important factors for the proper function of each physiological barrier. An imbalance in Ca^2+^ transport in senescent cells can result in reduced ERK1/2 phosphorylation and slower thrombin signaling in age-related barriers, thereby disrupting the endothelial barrier [143]. Ca^2+^ dysregulation in senescent cells may further activate p38 mitogen-activated protein kinase (MAPK), phosphatase, and protein kinase C (PKC), thereby disrupting TJs and gap junctions in each biological barrier and affecting their integrity and normal function [144]. Ca^2+^ can also participate in autophagy through various pathways, such as IP3R, beclin1, and βCaMKKβ-AMPK-mTOR [145]. The dysregulation of Ca^2+^ in the age-related barriers of senescent cells allows for abnormal cellular autophagy, which may be involved in the induction of abnormal cytotoxicity and barrier damage [144].

A defining characteristic of senescent cells is stable cell cycle arrest [146]. This cell cycle arrest is controlled by the activation of the p53/p21CIP1 and p16INK4a/Rb tumor suppressor pathways and renders senescent cells unresponsive to pro-division or growth factor stimulation and prevents them from re-entering the cell cycle, even under favorable growth conditions. These mechanisms highlight potential therapeutic targets for treating tumors [147]. SKN-SH glioblastoma occurs in children, and high concentrations of doxorubicin cross the BBB to inhibit p21 and induce SKN-SH apoptosis [148]. In addition, the loss of RB protein in senescent cells triggers the upregulation of p53 through ARF or DNA damage signaling in age-related barriers [149]. p53/ARF and RB/p16 block the tumor cell cycle and implement the senescence program in a synergistic and interconnected manner [150], thus preventing tumor cell escape from senescence and malignant transformation.

#### 3.6.2 Gene Expression and Secretory Phenotype

Despite growth cycle arrest, senescent cells are metabolically active and can affect the expression of various cellular gene profiles in barriers, thereby altering the cellular microenvironment and inducing an impaired barrier. In senescent cells, cell cycle inhibitors and cell cycle protein-dependent kinase inhibitors (CDKIs), including p21 (CDKN1a) and p16 (p16INK4a), are overexpressed, and genes encoding stimulatory cell cycle proteins are repressed, further driving cellular senescence and its accumulation and potentially accelerating barrier aging and destruction [151].

In a previous study using UVB to induce premature senescence in human skin fibroblasts, investigators found increased mRNA levels of the senescence-related genes, fibronectin, bone connexin, and SM22, along with the upregulated expression of cell cycle-related proteins, including p53, p21, p19, Hdm2, and Bax. There was also a decrease in the expression of BCL, HIF-1α, and vascular endothelial growth factor (VEGF), reflecting the possible involvement of senescent skin fibroblasts in the aging process of the skin barrier [152]. The researchers found that nuclear fibril laminin B1 (LMNB1) in senescent cells may be involved in the barrier aging process in at least two ways: the spatial reorganization of chronic proteins and genetic repression. LMNB1 in the central region of the riboflavin-associated structural domain (LAD) of senescent cells is disrupted during senescence, while the increased spatial re-localization of perinuclear H3K9me3-positive heterogeneous chromatin accelerates SAHF formation. Ectopic LMNB1 (outside of LAD) can inhibit this process [153].

Changes in cytokines, interleukins, chemokines, proteases, growth factors, degradative enzymes (matrix metalloproteinases [MMPs]), and soluble protein/extracellular matrix components in age-related barriers that affect protein expression and enable senescent cells to communicate with neighboring cells are referred to as the SASP or senescence-informed secretome. The SASP accelerates the expansion and proliferation of senescent cells by stimulating extracellular signals from proximal progenitor cells that promote tissue regeneration, accelerate the aging process of biological barriers, and contribute to suppressing tumorigenesis [154]. Fibroblasts in patients with Hutchinson-Gilford premature aging syndrome lose the H3K27me3 of mutant laminin A and upregulate the expression of key senescence genes [155]. In proliferating cells, lamin B1 is reduced, allowing proliferating cells to fail prematurely, which may indicate a potential therapeutic target for anticancer therapies [156]. H2A.J overexpression leads to the increased expression of SASPs in proliferating cells, leading to chronic inflammation, an impaired barrier, and the accelerated development of age-related diseases [157].

#### 3.6.3 ROS and Inflammation

Although senescent cells stop dividing, increased oxidative and endoplasmic reticulum stress due to increased cell volume, increased SASP protein production, elevated glycolysis, an imbalance in glycolytic enzyme activity, decreased mitochondrial respiration, and decreased ATP production leaves the cells in a stressed state because of a lack of energy and a ROS overproduction barrier when replicating in the senescent state [158]. These complications induce a stressful state at each biological barrier. Under normal physiological conditions, cells at biological barriers maintain a balance between the production and scavenging of oxygen-derived free radicals [159], or ROS. The imbalance between ROS production from senescent cells and the detoxification capacity of the biological system in the age-related barrier allows the production of ROS, including superoxide radicals (O2·-), hydrogen peroxide (H2O2), hydroxyl radicals (·OH), and singlet oxygen (1O2), as metabolic byproducts of the biological system [160]. When ROS levels exceed the antioxidant capacity of the cell, they react with lipids, proteins, and nucleic acids, leading to oxidation or peroxide production, disruptions in cell membrane structure, changes in permeability, and cytotoxic reactions, which cause changes to the integrity and permeability of each biological barrier [161]. ROS levels in cytotoxic T cells involved in the immune surveillance of cellular senescence have been found to increase with age, and the activation of interferons (IFNs), TNF, and inflammatory signaling increase, with each age-related barrier being more prone to inflammatory features [162]. T and B lymphocytes have high proliferative capacities and are highly susceptible to replicative senescence, which can impair the immune response. Each biological barrier plays an essential role in human immunity. The accumulation of senescent T and B cells on age-related barriers, which remain active and produce pro-inflammatory cytokines and active mediators of NK cells in large numbers, may induce the chronic low-grade inflammation of the age-related barrier, thereby disrupting its integrity and function [163].

The immune response is also a key factor driving the aging of physiological barriers. T lymphocytes in age-related barriers undergo senescence. This is accompanied by the high expression of p16 and p21; specific secretome features of interleukin-6 (IL-6), IL-8, IL-10, TGF, IFN, and TNF production; downregulation of surface markers (e.g., CD28 and CD27); and upregulation of PD1 [164]. A study involving SASP-related factors, such as TGF-β family ligands, CCL2, VEGF, and CCL20, in human fibroblasts revealed that inflammatory vesicles mediate IL-1 signaling and control SASP expression, which may be involved in slowing the rate and spread of cellular senescence [165]. The knockdown of the nfkb1 subunit of the mouse transcription factor, NF-κB, induces chronic, low-grade inflammation that leads to premature senescence in mice. In addition, chronic inflammation exacerbates telomere dysfunction and cellular senescence, leading to reduced regenerative potential in multiple tissues and accelerated aging [166]. Senescent tumor cells secrete excessive amounts of SASP-related factors, such as the pro-inflammatory cytokines, IL-6, IL-8, and IL-1. The expression levels of other chemokines that bind to the IL-8 receptor C-X-C motif chemokine receptor 2 (CXCR2), such as CXCL-2, CXCL-3, and CXCL-5, are further increased. The expression levels of ccl-2 (MCP-1), ccl-20 (MIP-3), ccl-7 (MCP-3), CXCL-4 (PF-4), CXCL 1 (Gro -), and CXCL-12 (SDF-1) are also significantly increased in replicative senescent cells [167], allowing the disruption of the blood-tumor barrier integrity and greater spread of tumor cells. Currently, IL-1 is considered one of the major regulators of the SASP. The release of IL-1 from senescent cells transmits senescence to normal and tumor cells, and IFN induces senescence and an impaired barrier by triggering DNA damage in target cells but inevitably accelerates blood-tumor barrier aging, thus leading to an impaired barrier [168].

Macrophages are key immune cells that protect the body from pathogens and are a critical immune cell type in the blood-tumor barrier [169]. The expression of senescence markers (p16INK4 and p14/p19ARF) in mouse bone marrow-derived macrophages, human adipose tissue macrophages, and pro-inflammatory macrophage-related genes are significantly downregulated and that of anti-inflammatory macrophage-related genes are significantly upregulated in p16INK4a-deficient bone marrow-derived macrophages [170]. This suggests that macrophages are also involved in pathological processes associated with cellular senescence-induced barrier damage.

#### 3.6.4 Anti-Apoptosis

Cellular senescence and apoptotic pathways are simultaneously involved in stress responses during age-related barrier dysfunction, and responses to barrier damage determine whether the cells in that barrier will undergo senescence or apoptosis [171]. Apoptosis occurs when cells encounter excessive stress, whereas senescence occurs in response to less severe injury. Low doses of adriamycin have been found to lead to senescence, and high doses lead to apoptosis in MCF7 breast cancer cells [172]. In addition, neonatal rat cardiomyocytes have shown similar dose-dependent responses to adriamycin, which led to DNA damage and senescence when administered in low doses and apoptosis when administered in high doses [173]. ROS damage exerted a similar effect, with high doses of H2O2 leading to apoptosis and low doses inducing senescence in F65 and IMR90 human diploid fibroblasts [174]. However, DNA damage can determine the cell response. The treatment of cells with any dose of DNA-damaging agents (e.g., leucovorin) causes senescence but not apoptosis [175]. In addition to the nature and extent of these stressors, the balance between cellular senescence and proapoptotic pathways determines the course of the biological barrier. Barrier aging or disruption due to the accumulation of cellular senescence is usually less severe than that caused by apoptosis because senescent cells remain metabolically active [176].

In mouse embryonic fibroblasts with p53 mutation-induced senescence rather than UVB-induced apoptosis, the expression of pro-apoptotic factors (PUMA and NOXA) is downregulated, and the pro-survival gene BCL-2 is highly expressed, allowing the PB to develop normally and supply fetal nutrition [44]. The PKC family is upregulated in MRC5 human lung fibroblasts undergoing irradiation-induced senescence, and the knockdown of PKC leads to decreased levels of BCL-2, phosphor-BAD, and phosphor-CREB, leading to significant p53-induced apoptosis and disruptions in blood-air barrier integrity and function [177]. Senescent cells further play various roles in cancer therapies. In some cases, apoptosis-protective genes may be expressed in conjunction with dominant transforming oncogenes, both of which are necessary for cancer. Researchers are optimistic that by inducing cellular senescence in tumor tissues, tumor cells may stop growing. In this scenario, the blood-tumor barrier and surrounding normal tissues would not undergo apoptosis to trigger excessive human autoimmunity [178].

In this section, we review the role of cellular senescence in age-related biological barriers and the mechanisms that induce cellular senescence and barrier impairment. Cellular senescence occurs not only in diseased barriers but also in normal physiological responses; therefore, senescent cells may be associated with the breakdown of biological barriers and the development of cellular senescence-associated barrier diseases.

## 4. Cellular Senescence, Age-Related Barriers, and Associated Barrier Diseases

Various biological barriers in the human body are key blocks that allow for communication between various organs and peripheral blood circulation. They also play an influential role in providing nutrients for human development and maintaining the homeostasis of the microenvironment. However, as individuals age, senescent cells gradually accumulate on the age-related barriers, and the functions of barriers begin to become dysregulated. This dysfunction or inability to respond to the needs of various organs may lead to the appearance of related diseases. This section describes several cellular senescence-associated barrier diseases in which the dysfunction of the barriers related to cellular senescence and age plays a major causal role. There are few studies on cellular senescence-associated barrier diseases at present, however, which indicates that this is a field that requires further research.

### 4.1 Alzheimer’s Disease

Aging is a major risk factor for neurodegenerative diseases, such as Alzheimer's disease. Alzheimer's disease is caused by the accumulation of psychopathic senescent cells with age, neurovascular units (NVU) dysfunction, and BBB impairment. Multiple tight and adhesive proteins are present in the BBB. With increasing age, endothelial cells and pericytes in the BBB undergo aging, the tight/adhesive junctions of the BBB are disrupted, and vascular permeability decreases, resulting in a reduction in the number of cells, NVU dysfunction, and BBB impairment with cerebral blood outflow and an elevation in brain amyloid-β levels [179][180].

Interactions between endothelial cells and pericytes are critical for BBB formation and maintenance [181]. In senescent endothelial cells and pericytes, vascular-associated MMP levels gradually increase and influence cell signaling during aging by regulating the expression and activity of cytokines, chemokines, growth factors, hormones, and angiogenic factors [182]. This process causes a decrease in the number of tightly linked adaptor molecules that adhere to junction proteins [183] and a significant increase in BBB upstream flow phagocytosis, which further accelerates BBB catabolism. This process causes the extravasation of large molecules, such as brain amyloid-β, into the brain parenchyma in large and small circulation territories [184]. The BBB regulates brain amyloid-β levels through multiple pathways. Receptors for advanced glycation end products (RAGE) and low-density lipoprotein receptor-related protein 1 (LRP1) are involved in Aβ transport and are elevated in expression in age-related BBB [185]. RAGE mediates Aβ expression on age-related BBBs. In neuronal cells and astrocytes with a senescent phenotype, RAGE expression is increased, disrupting age-associated BBB integrity, providing an opportunity for Aβ and Aβ-rich monocytes to cross BBB, reduced Aβ clearance in BBB, and accelerated deterioration in patients with Alzheimer's disease [186]. Under physiological conditions, LRP1 in endothelial cells binds to the Aβ in the proximal lumen of the BBB and reduces its blood concentration [187]. P-glycoprotein 1 (PG1) is a protein expressed in endothelial cells and mediates the flow of drugs and xenobiotic compounds through the endothelium into the blood in the brain. PG1 scavenges Aβ via LRP1 on the BBB, effectively preventing the accumulation of Aβ and other substances in the endothelium of the brain [188]. However, senescent endothelial cells are subjected to excessive ROS damage, and PG1 and LRP1 are not only reduced in number but also oxidized. Their activity is also drastically reduced, making PG1 and LRP1 unable to bind or transport Aβ, which is retained in the brain [189][190]. Increased APOE4 expression in endothelial cells blocks the blood clearance of Aβ by LRP1 [191]. The APOJ content in endothelial cells decreases, similar to the ability of Aβ in the brain to cross the BBB through LRP2 into the blood. As such, Aβ accumulates in the cerebral vasculature and brain during the aging process in patients with Alzheimer's disease [192].

### 4.2 Parkinson’s Disease

Parkinson’s disease is a neurodegenerative disease that mostly occurs in the elderly and is highly age-dependent. The prevalence of Parkinson’s disease is rapidly increasing in the global aging population [193]. Astrocytes perform various functions in the BBB, including providing support and immunity. In the age-related BBB, there is an increase in senescent astrocytes, collapse of the cell membrane of these cells, a decrease in support, and a corresponding change in antigen presentation capacity. It has been shown that major histocompatibility complex (MHC)-I expression by astrocytes on the BBB is upregulated with age in the normal aging population and that MHC-I exerts a protective effect on neurons and contributes to the maintenance of cognitive abilities in the elderly population. In contrast, MHC antigen expression in senescent astrocytes is upregulated in patients with Parkinson’s disease compared to that in the healthy aging population [194]. More senescent astrocytes are present in the age-related BBB, and MHC-II is highly expressed in senescent astrocytes and wraps around blood vessels, infiltrating CD4+ T cells, engaging in crosstalk with T cells, increasing the load of pathologically phosphorylated a-synuclein (aSYN), and accelerating the Parkinson’s disease process [195]. In the age-related BBB, there is an increased production of the astrocyte CXCL10, a peripheral immune cell sequestrant that causes T cells to adhere to vascular endothelial cells, which exacerbates the BBB immune response and advances the development of Parkinson’s disease [196].

A study assessing dynamic contrast-enhanced (DCE) magnetic resonance images of Parkinson’s disease patient brains revealed gadolinium BBB leakage in the basal ganglia of early-stage Parkinson’s disease, suggesting that BBB destruction in the basal ganglia may have occurred prior to basal ganglia degeneration [197] and that BBB integrity is critical for preventing Parkinson’s disease. The increase in the number of senescent endothelial cells and pericytes, decrease in the thickness, length, and density of endothelial cells, degeneration of microvessels, accumulation of inflammatory substances in the age-related BBB, and loss or premature decay of TJ proteins cause changes in the basement membrane, induce capillary leakage, and induce inflammatory changes in the brain tissue [198].

### 4.3 Age-Related Macular Degeneration and Diabetic Retinopathy

Age-related macular degeneration is a common chronic debilitating condition that results in ocular vision loss characterized by the early appearance of retinal neuroinflammation. Its prevalence is strongly correlated with increasing age [199]. Normal age-related BRB transforms into pathological age-related macular degeneration through vascular inflammation, vascular dysregulation, mitochondrial damage, ROS production, and RPE cell senescence. Age-related BRB is accompanied by Aβ accumulation in RPE cells, causing RPE cell vacuolization and damage, which may lead to RPE cell atrophy and/or the formation of RPE subdeposits that affect BRB function and lead to the loss of visual function [200]. After RPE injury, cells undergo epithelial-mesenchymal transition, which converts epithelial cells into myofibroblasts. RPE cell stress fibers divide focally and thicken, making the retina insufficiently pigmented and inducing early age-related macular degeneration [201]. Cone-shaped photoreceptors accumulate in the central macular recess and gradually degenerate with age, causing irreversible damage to the macula, resulting in the thinning of the macula and blurring of central vision, and leading to severe age-related macular degeneration [202]. Approximately 15% of patients with age-related macular degeneration exhibit an abnormal increase in senescent retinal endothelial cells, in which the balance of anti-angiogenic factors is altered, leading to the overproduction of VEGF and choroidal neovascularization (CNV), damaging the BRB, and affecting the central visual system [203][204].

The number of patients with and severity of diabetes is inextricably linked to disease duration, with typical complications including diabetic retinopathy [205]. Diabetic retinopathy is a microvascular disease of the eye caused by hyperglycemia. It damages the BRB and leads to vision loss or even blindness [206]. In the elderly, the prevalence of diabetic retinopathy is significantly higher than that in younger patients, and its severity is positively correlated with the duration of the disease [207], possibly due to the increased accumulation and spread of senescent cells in the BRB over time. With aging, senescent cells accumulate more in the age-related BRB, and changes in BRB metabolism occur. In senescent RPE cells and Bruch's membranes, advanced glycation end products accumulate, the production of methylated metabolites significantly increases, and VEGF is released, allowing a significant increase in ROS production and accumulation in pericytes and causing severe and irreversible damage, which leads to pericyte apoptosis in the BRB. Pericyte apoptosis is one of the first symptoms of age-related macular degeneration and diabetic retinopathy [208].

In healthy individuals, cellular glucose is processed by hexokinases in retinal cells, whereas hexokinase expression is reduced in senescent retinal cells [209]. In contrast, high glucose levels within the senescent retinal cells of patients with diabetic retinopathy increase the load on hexokinase, saturating it and shunting excess glucose to the polyol pathway [210]. Glucose is converted to sorbitol by aldolase reductase (AR), which consumes large amounts of NADPH, thereby reducing the function of glutathione reductase and impeding the inherent antioxidant activity within retinal cells. By contrast, sorbitol cannot be transported out of retinal cells and can only be slowly converted to fructose by sorbitol dehydrogenase (SDH), which is further phosphorylated to fructose-3-phosphate and converted to 3-deoxyglucosone, both of which are potent glycating agents that may lead to retinal osmotic damage or even cell death [211]. In senescent retinal cells, glutathione reductase activity is blocked, SDH activity is increased, which promotes ROS production, and AR activity is significantly upregulated, which may damage the age-related BRB [212]. AR levels are significantly higher in the retinal vascular systems of aged rats than in those of younger rats. Senescent retinal endothelial cells also have significantly lower antioxidant capacities, and senescent Müller cells have a higher risk of oxidative damage, which plays an important role in the development of diabetic retinopathy in older patients [213].

In age-related BRBs, senescent retinal cells accumulate, and metabolism is subsequently altered. The polyol and AGE pathways play important roles in the initiation of oxidative stress in senescent retinal cells, weakening the defense of age-related BRBs against oxidative stress. Accumulated VEGF in senescent retinal cells may also exacerbate the reduced blood flow in age-related BRBs. These pathways act synergistically and play important roles in the progression of age-related macular degeneration and diabetic retinopathy.

### 4.4 Preeclampsia

Cellular senescence caused by aging affects the cardiovascular, skeletal, and metabolic functions in women [214], particularly the female reproductive system. In addition, cellular senescence is involved in the regulation of menarche, reproductive lifespans, and menopause and has a more prominent impact on pregnancy and its complications, especially preeclampsia [215].

Preeclampsia is an age-related placenta-mediated multisystem hypertension in women with systemic inflammation, systemic endothelial dysfunction, oxidative stress surges, and vascular injury [216]. Pregnancy is a natural model of human maturation with the continuous appearance and accumulation of senescent cells in the PB, which leads to syncytial trophectoderm formation, placental implantation, embryonic development, and delivery [217]. In older pregnant women, cells in age-related PBs are more susceptible to metabolic stressors, such as oxidative stress, inflammation, DNA methylation, and telomere shortening, which further accelerate cellular senescence and maternal vascular aging [218]. Maternal age is an independent risk factor for preeclampsia and hyperemesis, and aging maternal vasculature on the PB allows abnormal placental implantation, leading to placental hypoxia and a significant increase in oxidative stress. These issues can accelerate the onset of placental aging and induce adverse pregnancy outcomes, such as preeclampsia or early onset preeclampsia, intrauterine growth restriction, and preterm delivery [219]. Preeclampsia also causes extensive maternal vascular endothelial damage that persists long after the end of the pregnancy [220]. The massive accumulation of senescent endothelial cells during and after preeclampsia leads to extensive endothelial dysfunction accompanied by increased oxidative stress, decreased endothelial nitrogen oxide (NO) bioavailability, increased endothelial-derived vasoconstrictor factors, and increased expression levels of pro-inflammatory cytokines, all of which leave normal endothelial cells in a state of prolonged repair damage, accelerate vascular endothelial cell senescence and damage to age-related PBs, and induce age-related psychological disorders in women [221].

DNA damage is associated with senescence and plays an important role in the cellular senescence of age-related PBs [222]. Investigators have found significantly increased levels of DNA damage in mononuclear leukocytes in patients with mild eclampsia and their offspring, which may lead to greater susceptibility to senescent monocytes. However, this damage was more confined to the maternal side of the placenta, suggesting that there may also be a link between DNA damage and preeclampsia [223]. The C3 complement also plays an important role in cellular senescence in age-related PBs and has been associated with severe preeclampsia. Elevated C3 expression in neurons in the brain of patients with severe preeclampsia is consistent with the cellular expression of senescent phenotype neurons in the brains and cerebrospinal fluid of patients with Alzheimer's disease [224]. Soluble fms-like tyrosine kinase 1 is an antagonist of VEGF and placental growth factor, and its expression is increased in senescent cells in aged mice, leading to an imbalance in angiogenesis, a typical feature of early preeclampsia [225].

**Figure 2.**
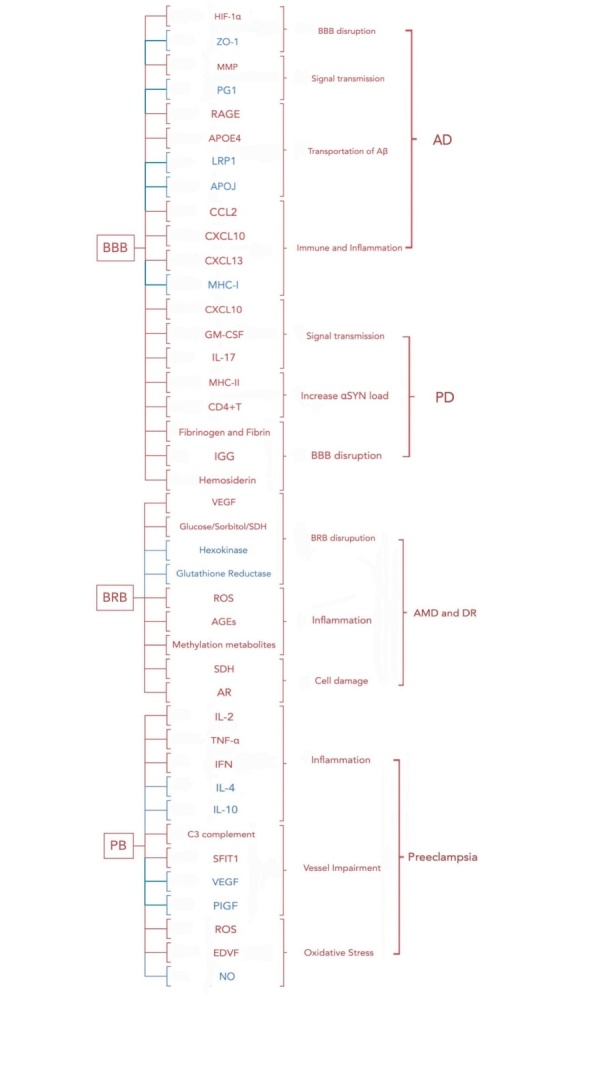
Factors involved in regulating age-dependent physiological barrier diseases and their pathological processes.

The integrity of the biological barrier is an important marker of human health, and damage to the mechanical barrier can disrupt the homeostasis and function of relevant systems and cause a wide range of pathogenicity [226]. Cellular senescence is inextricably associated with the integrity of age-related biological barriers. The accumulation and proliferation of large numbers of senescent cells may therefore lead to barrier disruption. In this section, we describe cellular senescence with age-dependent biological barriers and associated diseases, such as Alzheimer’s disease, Parkinson’s disease, and age-related macular degeneration, which have unique cellular senescence-associated disease processes (Fig. 2).

## 5. Intervention of Cellular Senescence and Associated Barrier Diseases

Cellular senescence is a natural process that plays a key role in the pathogenesis of several cellular senescence-associated barrier diseases, including age-dependent biological barrier diseases such as Alzheimer’s disease, Parkinson’s disease, and age-related macular degeneration. In this section, we provide the latest information on the interventions and treatments for senescence and cellular senescence-associated barrier diseases associated with aging. We focus on the role and potential mechanisms underlying these interventions in the regulation of senescence and the treatment of diseases.

### 5.1 Metformin

Metformin is the most widely used anti-diabetic drug. It regulates blood glucose by inhibiting hepatic gluconeogenesis and promoting insulin sensitivity to facilitate the cellular uptake of glucose and is commonly used in the treatment of patients with type 2 diabetes mellitus. Recent studies have shown that metformin also delays senescence and slows the progression of age-dependent diseases by affecting key senescence marker events, including the dysregulation of nutrient sensing, loss of proteostasis, mitochondrial dysfunction, and interstitial changes [227].

The dysregulation of nutrient-sensing pathways leads to a gradual deterioration in the regulation of metabolic homeostasis, which accelerates senescence [228]. At the molecular level, metformin affects human nutrient-sensing pathways by decreasing insulin, IGF, and mTOR1 expression and increasing AMPK and SIRT1 expression. Metformin inhibits insulin and IGF expression and the insulin receptor substrate 1/2 (IRS 1/2) and PI3K/AKT/mTOR pathway phosphorylation, thereby controlling cellular senescence [229]. Metformin also activates the IRS/PI3K/AKT pathway to improve metabolism and decrease cognitive impairment in elderly individuals [229]. Both mTOR1 and mTOR2 belong to the mTOR kinase family; however, only mTOR1 is involved in regulating the human lifespan. Metformin activates tuberous sclerosis in a Ras-related GTP-binding protein (Rag)- and GTPase-dependent manner or via the AMPK pathway complex2 (TSC2) pathway to inhibit mTOR1 expression [230]. Protein homeostasis is important for cell functionality and viability; however, senescence and related diseases are often accompanied by a loss of protein stability. Metformin has been found to regulate unfolded protein response-related chaperone proteins (UPRs), including HSP60, HSP90, glucose-regulated protein 78 (GRP78), and the C/EBP homologous protein, in a D-galactose-induced senescence rat model. Metformin further maintains proteostasis and mitigates age-related hearing loss and neurodegeneration [231]. In addition, metformin ameliorates inflammation by stimulating Krüppel-like factor 2 (KLF2) expression and autophagy in different cell types, such as T cells, endothelial cells, and macrophages [232]. These studies have suggested that metformin maintains protein stability by inhibiting protein misfolding and enhancing autophagy, thereby reducing senescence and related diseases [233]. Metformin also inhibits cellular senescence in SASPs and causes multiple age-related dysfunctions [234]. In lens, epithelial and nuclear pulposus cells, metformin has been found to reduce SASPs and senescence by activating AMPK signaling and restoring autophagic flow [235]. Metformin further inhibits endothelial senescence by activating SIRT1 activity and inhibiting macrophage senescence and SASPs by downregulating NLRC4 phosphorylation [236].

Metformin has significant anti-senescence and attenuating effects on diseases and has shown promise in age-related clinical applications. However, the molecular mechanisms underlying the reduction of senescence and age-dependent diseases are poorly understood and require further investigation.

### 5.2 Quercetin and Dasatinib

Quercetin [2-(3,4-dihydroxy phenyl)-3,5,7-trihydroxy-4H-1-benzopyran-4-one] is a natural, nontoxic flavonoid and lipophilic compound. It can be absorbed by simple diffusion across the intestinal membrane but is mainly ingested as a glycoside. Quercetin not only has many beneficial roles in neuroprotection but also has a specific role in age-dependent neurologic disorders [237].

Quercetin exerts antioxidative stress effects. By modulating the SIRT1/Nrf2/HO-1 pathway, quercetin protects neurons from oxidative stress damage induced by Aβ aggregation [238]. Quercetin also reduces oxidative stress-mediated hippocampal neuronal death by promoting the phosphatidylinositol 3-kinase (PI3K)/Akt-mediated downregulation of glycogen synthase kinase 3β (GSK-3β) activity [239]. Quercetin further exerts anti-inflammatory activity. Studies have shown that quercetin attenuates the production of inflammatory mediators, such as NO and TNF, and reduces the levels of inducible NO synthase (iNOS), thus exerting anti-neuroinflammatory effects on LPS-activated BV-2 microglia [240]. In contrast, quercetin upregulates SIRT1 activity to enhance the anti-inflammatory activity of NF-κB acetylation in nerve and glial cells and reduce neuronal death [241]. Quercetin has also been found to reduce mitochondrial dysfunction in APPswe/PS1De9 transgenic mice by activating AMPK, thereby improving cognitive function in chronic Alzheimer’s disease [242]. Quercetin also markedly alleviates chronic Alzheimer's disease by restoring the expression of cell cycle proteins (including cell cycle protein B located in the FoxO signaling pathway perturbed by Aβ accumulation) in *Drosophila* Alzheimer's disease models [243].

Quercetin also exerts antisenescence effects in Parkinson’s disease. In a 6-hydroxydopamine (6-OHDA) Parkinson’s disease rat model, quercetin attenuated the unilateral medial forebrain tract (meningeal lesions) and striatum (partial lesions) via antioxidant, anti-inflammatory, and protective neurotransmitter mechanisms [244]. Quercetin also ameliorated striatal dopamine depletion and 5-TH neuronal cell loss in a MitoPark transgenic chronic Parkinson’s disease mouse model [245]. Different doses of quercetin increased neuronal density by modulating the SIRT1/HO-1/Nrf2 pathway to reduce oxidative damage [246]. Quercetin downregulated pro-inflammatory cytokine expression in N9 microglia and reduced post-neuronal apoptosis in a Parkinson’s disease microglia (N9)-neuronal (PC12) co-culture system [247]. In aged mice, quercetin prevented microglial activation by modulating the SIRT1/NLRP3 pathway and inhibiting the interaction between the NLRP3 inflammasome and mitochondrial autophagy, thereby reducing neurotoxicity and neuroinflammation [248]. The researchers also found that quercetin (10 µM) stimulated CREB via PKD1 phosphorylation or regulated SIRT1/PGC-1α transcriptional activity via CREB generation in dopaminergic neuronal cell mitochondria, thereby upregulating BDNF gene expression in dopaminergic neuronal cells, leading to mitochondrial dysfunction in astrocytes, enhancing NLRP3 inflammasome activity, and promoting IL-1 expression. In an aged Parkinson’s disease rat model, quercetin acted as an autophagy enhancer and regulated the microenvironment, leading to neuronal death [249]. Quercetin further activates SIRT1, promotes autophagic PINK1 activation, reduces cytochrome c release, and activates cystatinase to maintain mitochondrial integrity, thereby preventing apoptosis [250]. Moreover, quercetin regulates the protein expression of Bcl2/Bax, release of cytochrome c, and nuclear translocation of apoptosis-inducing factor (AIF) [251].

Dasatinib, a tyrosine kinase inhibitor targeting c-KIT, SRC, and adrenergic receptors, can eliminate senescent human pre-adipocytes when used alone. The combination of dasatinib and quercetin (DQ) has been found to reduce the senescent cell load in naturally senescent and progesterone mice, improve cardiovascular function and carotid vascular reactivity in aging mice after being administered in a single dose, and reduce senescence markers in the extremities of mice exposed to radiation [252]. A clinical study was conducted in patients with diabetic nephropathy treated with dasatinib (100 mg/day) and quercetin (1000 mg/day) for 3 days. Paired adipose tissue, skin biopsies, and blood control studies collected from the patients before treatment and 14 days after starting treatment found a decrease in the senescent cells in the adipose and skin tissues and a decrease in the circulating SASP factors (IL-1α, IL-6, and MMPs) after brief DQ treatment [253]. These effects were superior to those found when administering the two drugs separately. The treatment of Alzheimer's disease transgenic mice expressing mutant human amyloid precursor protein (APP) and presenilin-1 (PS1) with the DQ detoxification cocktail selectively eliminated senescent cells and reduced neuroinflammation while improving cognitive deficits [254].

### 5.3 BCL-2 Inhibitors

Navitoclax is a BH3 mimetic and a BCL family inhibitor of triple Bcl-2, Bcl-xL, and Bcl-w. Navitoclax exerts its antiaging activity mainly by inhibiting Bcl-w and Bcl-xL [255]. A study on the treatment of human mesenchymal stromal cells with navitoclax revealed a moderate anti-senescence effect, with reduced SA-β-Gal staining. However, further measurements of telomere length and epigenetic senescence characteristics revealed that navitoclax did not enable the regeneration of the mesenchymal stromal cells [256]. After navitoclax in mice treated with tislelizumab-based chemotherapy, senescent tumor cells were significantly removed, and the risk of tumor recurrence was reduced [257]. In addition, navitoclax appeared to be active in select senescent cells. Similarly combined siRNAs targeting Bcl-2, Bcl-xL, and Bcl-w induced the death of senescent HUVECs and IMR-90 cells in a study on human lung fibroblasts, senescent HUVECs, and pre-adipocytes [31]; however, there was no similar effect on the pre-adipocytes. In other melanoma and non-small cell lung cancer models, navitoclax has been found to selectively kill senescent tumor cells [258]. In TP53 wild-type breast carcinoma cells, navitoclax had no impact on cell proliferation but selectively induced apoptosis in a fraction of cells pre-treated with chemotherapy. Senescent breast carcinoma cells depend on Bcl-xL and/or Mcl-1 for survival. *In vivo* models of breast carcinomatosis have shown that the addition of navitoclax after chemotherapy improves tumor degeneration, increases survival in mice, and increases apoptosis [259].

There are concerns regarding the toxicity and side effects of navitoclax. Navitoclax has been shown to result in dose-limiting thrombocytopenia and neutropenia in patients [260]. Recently, researchers have proposed the use of galactose-encapsulated nanoparticles, a pre-drug galactose-fixed navitoclax (Nav-Gal) that can be preferentially activated by the SA-SAGal activity found in senescent cells, thereby increasing lysosomal and galactosidase activity in senescent cells, enabling the hydrolysis of cleavable galactose, and promoting the targeting of navitoclax to senescent cells. Compared to navitoclax, Nav-Gal is more specific in inducing cell death in senescent cells while protecting non-senescent cells. In the therapeutically relevant dose range, Nav-Gal induces significantly less thrombocytopenia in isolated human and mouse blood samples and *in vivo* than navitoclax [261].

Other researchers have similarly transformed navitoclax into a Bcl-xL protein hydrolysis-targeted chimera (PRO-TAC), PZ15227, which targets Bcl-xL to a cereblon (CRBN) E3 ligase for degradation. pZ15227 effectively clears senescent cells. However, because CRBN is poorly expressed in platelets, PZ15227 is less toxic to platelets, does not cause severe thrombocytopenia, and rejuvenates tissue stems and progenitor cells from aging mice [262].

**Table 1:** The Interventions of Cellular Senescence and their Possible Mechanisms.

Interventions	Possible Mechanism	References
**Increase the expression of tight junction proteins**	Protection of biological barriers disruption	54, 86
**Reduce IGG extravasation**	Protection of blood brain barrier disruption	55
**Inhibit the degradation of tight junction by reducing the expression of APOE4**	Protection of blood brain barrier disruption	275, 192
**Upregulate the expression of endothelial gap junction protein Gja1**	Protection of biological barriers disruption	135
**Reduce the expression of RAGE**	Protection of blood brain barrier disruption	186
**Upregulate the number of LRP1 and PG1**	Protection of blood brain barrier disruption	191
**Reduce ROS accumulation and upregulate NO bioavailability**	Protection of placental barrier disruption	222
**Regulating the expression of sFlt1 and promoting the role of VEGF and PlGF**	Protection of placental barrier disruption	226
**Decrease the expression of CCL4 chemokines in aging microglia**	Anti-inflammatory effect	72
**Upregulat the response of TNF-β and CSF-1, etc,. inflammation regulatory signals**	Anti-inflammatory effect	71
**Reduce the expression of upd inflammatory cytokines, making p38/Duox unable to be activated**	Anti-inflammatory effect	110
**Reduce the expression of IGF-1 and B2M**	Neuronal protection effect	62
**Upregulate the expression of GLAST and purinergic receptors**	Neuronal protection effect	77
**Upregulate the expression of MHC-I, downregulate MHC-II**	Neuronal protection effect	195, 196
**Downregulate C3 complement**	Neuronal protection effect	225
**Upregulation of MCU, Ca2+ signaling hotspot ERC and ITPR2**	Anti-calcium-regulation disorder	65, 66, 68
**Reduce the number and structure of MERCs**	Anti-calcium regulation disorder	67
**Reduce chronic accumulation of ROS to prevent disruption of normal Nr2 signals**	Regulation of JAK/STAT pathway	110
**Reduce ROS accumulation to reduce MPCs genetic chromatin loss**	Effect of regulating genetic expression and Secretory Phenotype	118
**Upregulate the expression of HMGB2**	Effect of regulating genetic expression and Secretory Phenotype	128
**Decrease the mRNA levels of fibronectin, osteointegrin and SM22**	Effect of regulating genetic expression and Secretory Phenotype	153
**Reducec the expression of LMNB1 in LAD, increase LMNB1 besides the area**	Effect of regulating genetic expression and Secretory Phenotype	154
**Inhibite the activation of p53/p21CIP1 and p16INK4a/Rb pathways**	Protect cell structure and cycle	148
**Upregulate the scavenging activity of O2%-, H2O2, %OH and 1O2**	Anti-oxidation effect	161
**Downregulate AR expression**	Anti-oxidation effect	212
**Overexpression of PKC to increase levels of BCL-2, phosphate BAD, and phosphate CREB**	Anti-apotosis effect	178
**Downregulate AGEs expression, reduce the accumulation of methylation metabolites in RPE and Bruce's membrane, and downregulate VEGF and ROS**	Anti-apotosis effect	209

### 5.4 Rapamycin

The hallmark of cellular senescence is the proliferation-like activity of growth-promoting pathways (e.g., mTOR and MAPK) in non-proliferating cells. When the cell cycle stalls, these pathways translate into senescence. Rapamycin is a reversible growth inhibitor that slows mTOR-driven cellular senescence [263].

The NIA intervention test program has been used to determine the effects of rapamycin on the lifespans of mice. Rapamycin (14 ppm or approximately 2.24 mg/kg), based on the average food consumption of the mice, extended their lifespans. Extension of life was also observed in mice administered rapamycin later in life (19 months) [264]. Moreover, rapamycin was effective over a wide range of doses and did not negatively affect the lifespans, even at high doses. The anti-senescence effects of rapamycin were not sex-specific; rapamycin extended the lifespans of both the male and female mice [265]. The increases in the lifespans of the female mice administered low doses of rapamycin were greater than those in the lifespans of the male mice; however, with high doses of rapamycin, the difference was small [266]. The number of senescent cells increases with age; therefore, cellular senescence may be an important mechanism in aging [267]. Although rapamycin was shown to extend the cellular lifespan, researchers began to investigate whether it affected cellular senescence. Rapamycin not only inhibited the expression of senescence markers, such as p16, p21, and SA-and Gal-positive cells but also reduced the SASP and expression and secretion of pro-inflammatory cytokines in senescent cells [268]. Researchers transplanted PSC27 senescent cells containing PC3 prostate cancer cells into SCID mice, treated the senescent cells with rapamycin prior to transplantation and reported a 50% reduction in the number of neoplastic prostate cancer cells [269]. Bleomycin is a common drug used in the treatment of oncological diseases; however, it tends to induce senescence in idiopathic pulmonary fibrosis in lung epithelial cells. The addition of rapamycin to a system co-cultured with bleomycin-treated cells and lung fibroblasts resulted in the attenuated proliferation of lung fibrogenic cells, reduced broblasts, attenuated proliferation of blasts, and reduced expression of SASPs by senescent cells [270]. K5rtTA/tet-Wnt mice exhibited progressive premature hair loss and epithelial stem cell senescence following the persistent expression of Wnt1 in the epithelial compartment of the skin. The treatment of the mice with rapamycin (4 mg/kg intraperitoneally for 18 days) reduced the accumulation of senescent epithelial stem cells and prevented Wnt1-induced hair loss in the K5rtTA/tet-Wnt mice for a prolonged period [271].

Rapamycin also affects age-dependent central nervous system disorders. Researchers administered rapamycin to transgenic Alzheimer's disease mice. The mice that received rapamycin for 2-3 months did not lose memory when they were approximately 6 months old [272]. The long-term rapamycin treatment of aged 3xTg-AD mice prevented tau pathology (tau phosphorylation) and protected cognitive ability, with no significant differences being observed compared to those in aged wild-type mice [273]. Furthermore, rapamycin reduced the accumulation of Aed tau pathology (tau phosphorylation), protected cmTOR signaling, and induced autophagy [47]. In APOE4 transgenic mice, rapamycin improved CBF, BBB integrity, and cognitive deficits [274]. Rapamycin treatment in different mouse models of Parkinson’s disease prevented the loss of tyrosine hydroxylase (TH+) neurons in the substantia nigra densa and improved muscle coordination, with excellent preventive effects [275].

Cellular senescence and age-dependent barrier diseases are physiological or pathological processes involving multiple tissues and organs in the human body. Therefore, multiple interventions exist (Table 1). This section assesses drugs that are widely used to intervene in aging or have exhibited clear efficacy in treating age-dependent barrier diseases, thereby delaying the initiation or progression of senescence in multiple ways.

## 6. Summary

Various physiological barriers in the human body play significant roles in the maintenance of internal environmental homeostasis. However, with aging and various environmental stressors, senescent cells gradually accumulate, the structural integrity of various cells on biological barriers is damaged, and the cell cycle is stalled, playing a role in anti-apoptosis. The SASPs secreted by senescent cells further accelerate cellular senescence and induce the aggravation of oxidative stress and related inflammation. Different degrees of aging in biological barriers occur, which we refer to as “cellular senescence-induced impaired barriers”. This occurs not only during normal aging but also in certain pathological conditions of the human body. Thus, cellular senescence-induced barrier impairment is closely associated with age-dependent barrier diseases in humans. Alzheimer's disease and Parkinson’s disease are age-related BBB diseases, the severity of which increases significantly with age-related BBB destruction. Similarly, age-related macular degeneration and diabetic retinopathy are associated with age-related BRB, and preeclampsia is associated with PB. Current anti-senescence measures mainly involve pharmacological interventions. Traditional anti-senescence drugs include D+Q and BCL-2 inhibitors; however, drugs for therapeutic-related diseases and with anti-senescence effects, such as metformin and rapamycin, have also been identified and primarily target anti-oxidative stress and inflammation. Clinical studies have shown significant effects of the drugs on lifespans. Each biological barrier in the human body is closely related to health, and with global aging trends, there is an urgent need to study cellular senescence-induced barrier impairment. Although there is a research basis for cellular senescence and related diseases, the understanding of cellular senescence-induced impaired barriers and related diseases remains limited, and the exact mechanisms underlying these conditions remain unclear owing to the lack of systematic research. Interventions are still limited to the anti-cellular senescence pathway, and there are no clear barriers for age-related target drugs for further investigation.
